# 2-Acetamidophenol (2-AAP) Suppresses the Progression of Atherosclerosis by Alleviating Hyperlipidemia and Attenuating the Ferroptosis Pathway

**DOI:** 10.3390/md22110513

**Published:** 2024-11-13

**Authors:** Xiaohan Zang, Yongcheng Wang, Cong Han, Lishuang Cui, Haojie Liu, Shuimiao Tian, Kechun Liu, Peihai Li, Chen Sun, Qing Xia, Yun Zhang

**Affiliations:** Biology Institute, Qilu University of Technology (Shandong Academy of Sciences), Jinan 250103, China; s15966519507@163.com (X.Z.); 19546143397@163.com (Y.W.); hcong0109@163.com (C.H.); 15369539412@163.com (L.C.); 17638439006@163.com (H.L.); 19906409460@163.com (S.T.); hliukch@sdas.org (K.L.); lipeihaihg@sina.com (P.L.); mornings0123@163.com (C.S.)

**Keywords:** atherosclerosis, 2-acetamidophenol, zebrafish, transcriptome analysis, ferroptosis

## Abstract

Hyperlipidemia and consequent endothelial inflammation, along with foam cell generation, promote the progression of atherosclerosis (AS). Here, we aimed to investigate the effects of 2-acetamidophenol (2-AAP), which was selected by zebrafish phenotypic screening, in alleviating AS by relieving hyperlipidemia and inhibiting foam cell formation, as well as the underlying mechanisms. In a zebrafish hyperlipidemia model, 2-AAP increased lipid-lowering efficacy; alleviated TC, TG, LDL-C, and MDA levels; elevated HDL-C and T-SOD levels; significantly improved intravascular macrophage aggregation; and improved blood flow. In an ox-LDL-induced RAW264.7 model, 2-AAP inhibited lipid phagocytosis in RAW264.7 cells; reduced the intracellular TC, TG, FC, and CE contents; and decreased the CE/TC ratio, thus slowing foam cell generation. In addition, 2-AAP alleviated intracellular ROS and ferrous ion accumulation in RAW264.7 cells, reduced the MDA content, and increased GPX4 viability. Furthermore, transcriptome analyses and gene expression validation showed 2-AAP treatment upregulates genes related to GSH synthesis and transport, such as gclc, gclm, gss, and gpx4a, and enhanced the expression levels of genes involved in the storage and transportation of iron ions, such as fpn1, fth, and g6pd, indicating that 2-AAP dramatically regulated the ferroptosis and glutathione metabolic pathways. Overall, our study demonstrated that 2-AAP demonstrated potential in AS by alleviating hyperlipidemia and attenuating the ferroptosis pathway and provided evidence supporting the future application of 2-AAP in AS treatment.

## 1. Introduction

Cardiovascular disease (CVD) triggered by atherosclerosis (AS) affects approximately 330 million people in China [1]. AS is characterized as a chronic inflammatory disease due to the initial accumulation of lipids in the walls of arterial vessels [2,3,4]. Hyperlipidemia, a major risk factor for AS, is a metabolic disorder that typically manifests as abnormally elevated levels of lipids or lipoproteins in the blood, accompanied by a systemic inflammatory response and oxidative stress [5]. Excessive lipid content in the blood affects the stability of blood flow, leading to lipid deposition on the surface of the vessel wall and impairing endothelial function [6]. High lipid content in the blood is an important predisposing factor for AS, and a zebrafish hyperlipidemia model induced by high-fat feeding was used in our preliminary experiments to screen for compounds with lipid-lowering effects as a means of searching for promising compounds with anti-AS activity. Moreover, in the high-fat state, cellular reactive oxygen species (ROS) production is increased, and LDL deposited beneath the endothelium is oxidized to form oxidized low-density lipoprotein (ox-LDL), which further impairs endothelial function [7]. Ox-LDL also upregulates the expression of dysfunctional endothelial cell adhesion molecules and chemokines, leading to persistent macrophage accumulation and inflammation [8].

Inflammatory response, lipid metabolism, endothelial dysfunction, and oxidative stress have been well described in AS [9]. In recent years, the role of ferroptosis in AS has attracted much attention. Ferroptosis as a cell death mechanism different from traditional apoptosis provides a novel target for the treatment of AS by affecting vascular endothelial cell function, macrophage polarization, and smooth muscle cell phenotypic switching [10,11]. Ferroptosis is characterized by the accumulation of reactive oxygen species (ROS) and lipid peroxides caused by increased levels of ferrous iron [12]. High intracellular iron levels promote macrophage differentiation toward the proinflammatory M1-type, which is the main cell type that phagocytose lipids to form plaques [13]. Glutathione peroxidase (GPX4) is a cellular antioxidant enzyme that plays an important role in stabilizing AS plaques by scavenging phospholipid peroxides from membranes [14]. Therefore, the viability of GPX4 is considered a marker of ferroptosis [15].

N-(2-hydroxyphenyl)-acetamide, also known as 2-acetamidophenol (2-AAP), is a secondary metabolite of sponge-derived actinomycetes [16]. A few relevant studies have been conducted, mainly in the direction of anti-osteoarthritis and antitumor uses [17,18,19,20,21]. In our previous article, we isolated 2-AAP from *Penicillium chrysogenum* Y20-2 and found its pro-angiogenic activity [22]. Furthermore, we screened marine-derived compounds for hypolipidemic activity using a zebrafish hyperlipidemia model and preliminarily found that 2-AAP showed a significant lipid-lowering effect. Considering the key role of lowering blood lipids in the treatment of atherosclerosis, we hypothesized that 2-AAP might have an anti-atherosclerosis effect. Here, we investigated the hypolipidemic, anti-inflammatory, antioxidative stress, and inhibitory effects of 2-AAP on foam cell formation and the corresponding underlying mechanisms to provide evidence supporting the future application of 2-AAP in the treatment of AS.

## 2. Results

### 2.1. 2-AAP Improved Lipid Metabolism in Zebrafish

**Figure 1:**
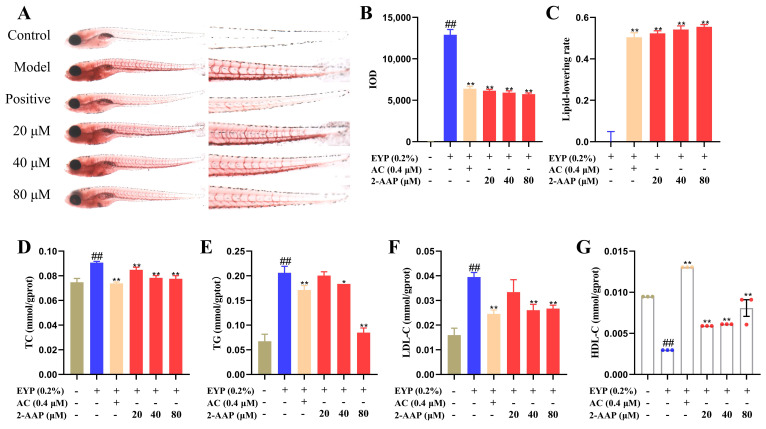
Effects of 2-AAP treatment on lipid metabolism in zebrafish after egg yolk powder feeding. (**A**) Typical images of zebrafish stained with Oil Red O. The whole fish was shown on the left, and the enlarged fish tails were shown on the right. (**B**) IOD values of Oil Red O staining in the tail, *n* = 10. (**C**) The lipid lowering rate of 2-AAP treatment, *n* = 10. Effects of 2-AAP treatment on TC (**D**), TG (**E**), LDL-C (**F**), and HDL-C (**G**) levels. In the column chart, brown represents the blank group, blue represents the model group, and red represents the administration group. Compared with the control group, ## *p* < 0.01. Compared with the model group, * *p* < 0.05, ** *p* < 0.01.

The vascular lipids of the zebrafish were stained with the lipid soluble dye Oil Red O. The results showed that, in comparison with the control group, the integrated optical density (IOD) in the caudal vasculature of the zebrafish in the model group was significantly greater, indicating that the administration of egg yolk powder increased the blood lipid content of the zebrafish. In comparison with the model group, the administration of atorvastatin calcium (AC) and 2-AAP markedly diminished the IOD, indicating enhanced lipid-lowering efficacy. Treatment with 80 μM of 2-AAP can achieve a lipid-lowering rate of 0.55. These findings suggest that 2-AAP may be an effective chemical for improving blood lipid levels in zebrafish (Figure 1A–C). Moreover, compared with the model group, treatment with 80 μM of 2-AAP resulted in triglyceride (TG, 0.085 ± 0.005 versus 0.206 ± 0.008 [mean ± SEM]; *p* < 0.01) and total cholesterol (TC, 0.091 ± 0.001 versus 0.078 ± 0.002 [mean ± SEM]; *p* < 0.01). Additionally, low-density lipoprotein cholesterol (LDL-C, 0.027 ± 0.001 versus 0.040 ± 0.002 [mean ± SEM]; *p* < 0.01) contents were decreased in a dose-dependent manner, and high-density lipoprotein cholesterol (HDL-C) was increased in a dose-dependent manner in the 20–80 μM 2-AAP groups (Figure 1D–G). In summary, 2-AAP effectively improved lipid metabolism.

### 2.2. 2-AAP Improved Blood Flow Velocity and Stability

**Figure 2:**
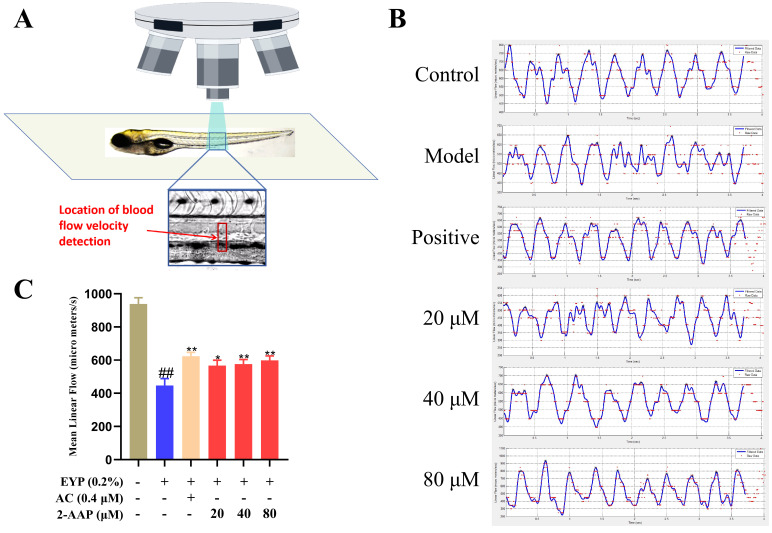
2-AAP improves blood flow velocity and stability. (**A**) Schematic diagram of the location of blood flow velocity detection. (**B**) Real-time detection of zebrafish blood flow velocity waveforms. (**C**) Mean blood flow velocity in the tail vessels of zebrafish, *n* = 7. In the column chart, brown represents the blank group, blue represents the model group, and red represents the administration group. Compared with the control group, ## *p* < 0.01. Compared with the model group, * *p* < 0.05, ** *p* < 0.01.

The shear stress exerted on the vessel wall is determined by two factors: blood flow rate and viscosity. When shear stress is low or oscillating, it impairs the repair function of endothelial cells in blood vessels, thereby promoting the development of atherosclerosis. The velocity of blood flow in zebrafish was measured with a microscope (Figure 2A). The real-time detection of zebrafish blood flow velocity waveforms revealed that egg yolk powder feeding could influence the stability of blood flow (Figure 2B). Furthermore, 2-AAP improved blood flow stability in zebrafish. Statistics on the average blood flow velocity in the caudal vessels of zebrafish revealed that the blood flow velocity in the caudal arteries of model zebrafish was lower than that in the control group. AC was observed to increase the blood flow velocity, and 20–80 μM 2-AAP accelerated the blood flow velocity. Compared with the model group, the blood flow velocity in the 80 μM 2-AAP groups increased by 1.3 times.

### 2.3. 2-AAP Improved Vascular Inflammation

**Figure 3:**
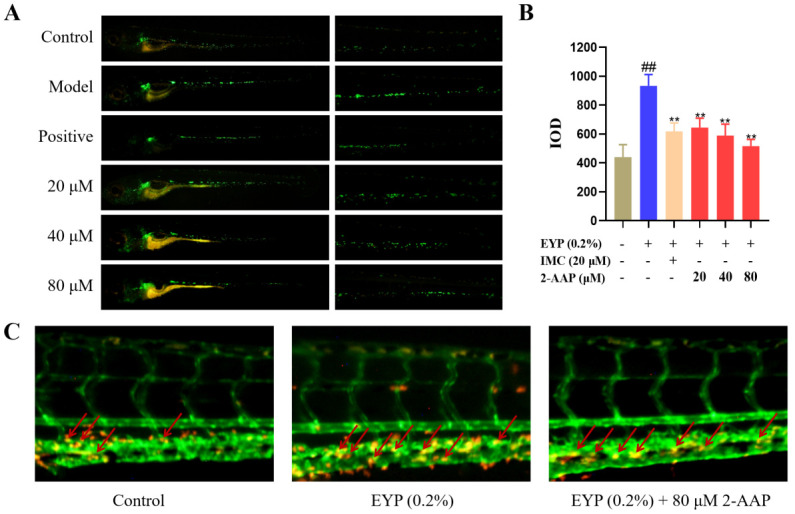
2-AAP improves vascular inflammation. (**A**) Phenotypic diagram of decreased macrophage density caused by 2-AAP. The whole fish was shown on the left, and the enlarged fish tails were shown on the right. (**B**) IOD values of immune cells that accumulated in the tail, *n* = 12. (**C**) Phenotypic diagram of alleviation of intravascular macrophage aggregation by 2-AAP. The red arrow indicates intravascular macrophages. In the column chart, brown represents the blank group, blue represents the model group, and red represents the administration group. Compared with the control group, ## *p* < 0.01. Compared with the model group, ** *p* < 0.01.

The inflammatory response plays a pivotal role in the pathogenesis of atherosclerosis, particularly within the vasculature. It can intensify damage to cells in the lining of blood vessels and accelerate the progression of atherosclerosis. This experiment utilized *Tg*(*lyz:EGFP)* zebrafish to quantify the density of macrophages following drug treatment. The findings revealed that 20–80 μM 2-AAP markedly diminished the macrophage density in zebrafish after high-fat feeding, with the effect exhibiting a concentration-dependent profile (Figure 3A,B). Macrophage density decreased by 46% in the 80 μM 2-AAP group compared to the model group.

Hybrid zebrafish with simultaneous dual fluorescence between the *Tg(fli1:EGFP)* and *Tg(lyz:DsRed2)* strains were used to observe intravascular macrophage aggregation. As shown in Figure 3C, high-fat feeding resulted in the aggregation of macrophages in blood vessels. The administration of 80 μM 2-AAP was efficacious in alleviating macrophage aggregation in blood vessels; thus, it can be postulated that 2-AAP offers the potential to mitigate vascular inflammation caused by elevated blood lipids.

### 2.4. 2-AAP Improved Oxidative Stress

**Figure 4:**
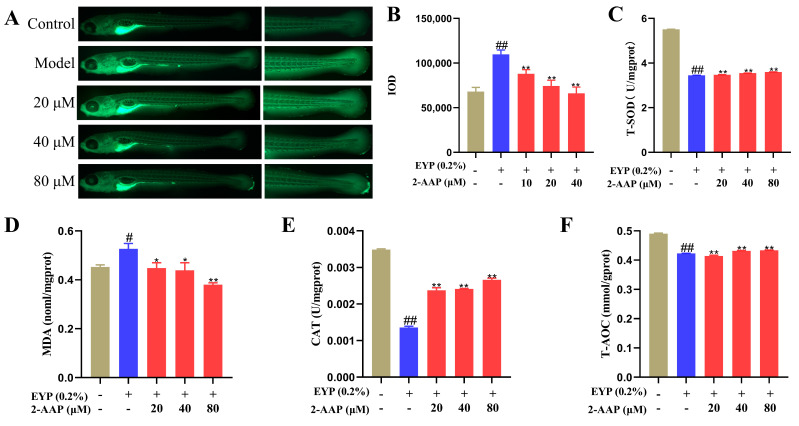
2-AAP improves oxidative stress. (**A**) Phenotypic diagram of reduced ROS fluorescence intensity caused by 2-AAP. The whole fish was shown on the left, and the enlarged fish tails were shown on the right. (**B**) IOD values of ROS accumulated in the tail, *n* = 16. Effects of 2-AAP treatment on T-SOD (**C**), MDA (**D**), CAT (**E**), and T-AOC (**F**) levels. In the column chart, brown represents the blank group, blue represents the model group, and red represents the administration group. Compared with the control group, # *p* < 0.05, ## *p* < 0.01. Compared with the model group, * *p* < 0.05, ** *p* < 0.01.

Zebrafish were incubated with the DCFH-DA probe: if the DCFH-DA probe bound to ROS in zebrafish, the probe would exhibit green fluorescence when photographed under a Zeiss fluorescence microscope, as shown in Figure 4A. The IOD statistics of the green fluorescence of zebrafish caudal blood vessels revealed a significant increase in the IOD in the model group compared with the control group. Conversely, 2-AAP decreased the IOD in a dose-dependent manner compared with the model group. Superoxide dismutase (SOD) and catalase (CAT) are key antioxidant enzymes in the body, and these enzymes scavenge free radicals in the body to protect the organism from oxidative stress damage. Whole homogenates of zebrafish were prepared to assay the activity of T-SOD and CAT and the total antioxidant capacity (T-AOC) (Figure 4C,E,F). 2-AAP restored T-SOD viability and T-AOC in a dose-dependent manner. The CAT activity of zebrafish was increased by two times when the concentration of 2-AAP was 80 μM. These results demonstrated that antioxidant enzyme activity in zebrafish was significantly increased following intervention with 2-AAP, indicating that 2-AAP was capable of significantly enhancing the antioxidant capacity of zebrafish. Lipids undergo peroxidation, and malondialdehyde (MDA) is the final oxidation product. The level of MDA was quantified in the model group and found to be significantly increased. Conversely, 80 μM 2-AAP significantly reduced MDA levels in vivo relative to the model group (0.380 ± 0.007 versus 0.527 ± 0.022 [mean ± SEM]; *p* < 0.01). Therefore, 2-AAP has good antioxidant activity.

### 2.5. 2-AAP Reduced the Phagocytosis of Oxidized Lipids in RAW264.7 Cells

**Figure 5:**
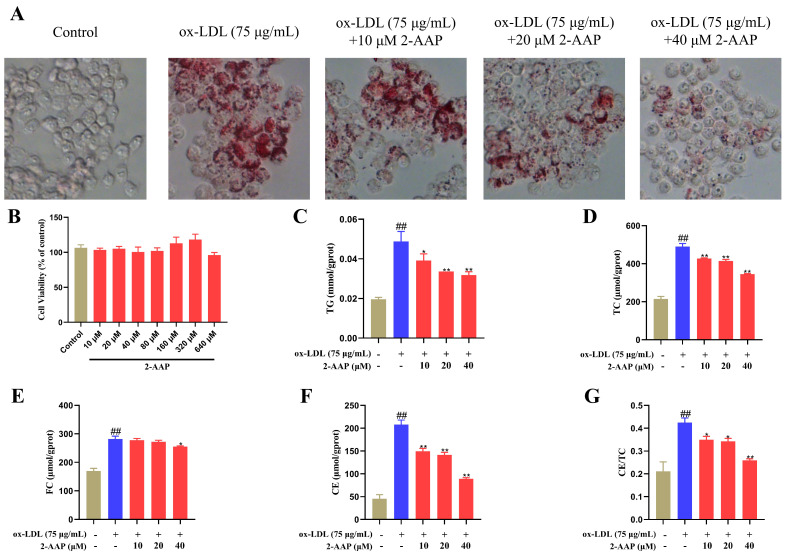
2-AAP reduces macrophage phagocytosis of oxidized lipids. (**A**) Oil Red O staining of macrophages. (**B**) Viability of RAW264.7 cells treated with 2-AAP. TG (**C**), TC (**D**), FC (**E**), CE (**F**), and CE/TC (**G**) contents in macrophages. In the column chart, brown represents the blank group, blue represents the model group, and red represents the administration group. Compared with the control group, ## *p* < 0.01. Compared with the model group, * *p* < 0.05, ** *p* < 0.01.

Owing to the lack of scavenger receptors on the surface of macrophages, when too many oxidized lipids are accumulated, macrophages differentiate into foam cells. The accumulation of foam cells subsequently leads to the formation of atherosclerotic plaques. As shown in Figure 5B, the administration of 2-AAP at concentrations ranging from 10 to 320 μM did not result in any discernible effect on cell viability. RAW264.7 cells were induced to phagocytose lipids with 75 μg/mL ox-LDL. The results revealed that the degree of Oil Red O staining in 2-AAP cells was lower than that in the model group (Figure 5A). These findings indicated that 2-AAP diminished the phagocytosis of ox-LDL by RAW264.7 cells. In addition, 2-AAP has the theoretical potential to reduce the intracellular TG, TC, free cholesterol (FC), and cholesterol ester (CE) contents, lowering the value of CE/TC (Figure 5C–G). As a result, 2-AAP could significantly inhibit the phagocytosis of oxidized lipids in macrophages and impede the formation of foam cells.

### 2.6. 2-AAP Improved Oxidative Stress and Ferroptosis

**Figure 6:**
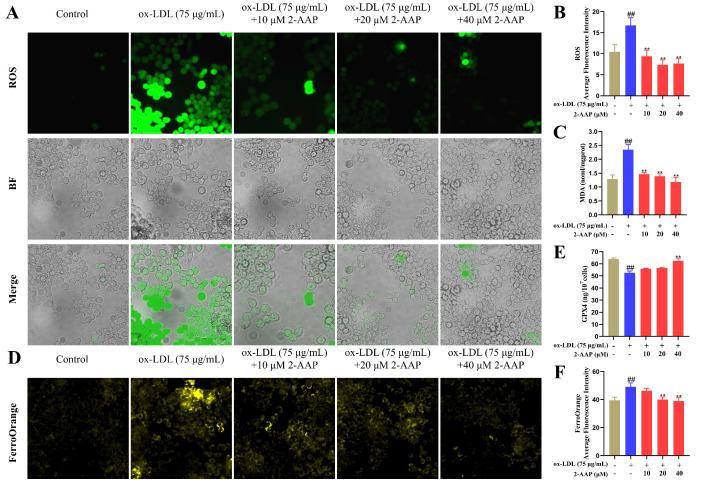
2-AAP improves oxidative stress and ferroptosis. (**A**) Typical diagram of ROS probe binding. (**B**) Statistics of the average fluorescence intensity of ROS. (**C**) MDA contents in the macrophages. (**D**) A typical FerroOrange diagram. (**E**) GPX4 vitality in macrophages. (**F**) Statistics of the average fluorescence intensity of FerroOrange. In the column chart, brown represents the blank group, blue represents the model group, and red represents the administration group. Compared with the control group, ## *p* < 0.01. Compared with the model group, ** *p* < 0.01.

We used an ROS-binding probe to investigate the antioxidant effects of 2-AAP in ox-LDL-treated RAW264.7 cells. As clearly shown in Figure 6A,B, treatment with 75 μg/mL ox-LDL resulted in significantly increased intracellular ROS levels, and the administration of 2-AAP inhibited the accumulation of intracellular ROS. A kit assay of intracellular MDA levels revealed that the phagocytosis of ox-LDL by RAW264.7 cells significantly increased MDA levels and that different 2-AAP concentrations reduced MDA levels in a dose-dependent manner (Figure 6C). Additionally, a double-antibody sandwich assay for GPX4 viability indicated that ox-LDL decreased intracellular GPX4 viability and that 2-AAP inhibited the decrease in GPX4 viability (Figure 6E). The fluorescent labeling of intracellular ferrous ions using a ferrous ion probe revealed that ox-LDL was able to induce elevated levels of ferrous ions in RAW264.7 cells compared with the blank group, and treatment with 2-AAP significantly reduced the number of intracellular ferrous ions compared with that in the model group (Figure 6D,F). Changes in ferrous ion levels and GPX4 activity, which are characteristic indicators of ferroptosis, suggested that 2-AAP might slow the atherosclerotic process by regulating ferroptosis and glutathione metabolism.

### 2.7. Results of Transcriptomic Analysis

#### 2.7.1. Results of Differential Gene Expression

**Figure 7:**
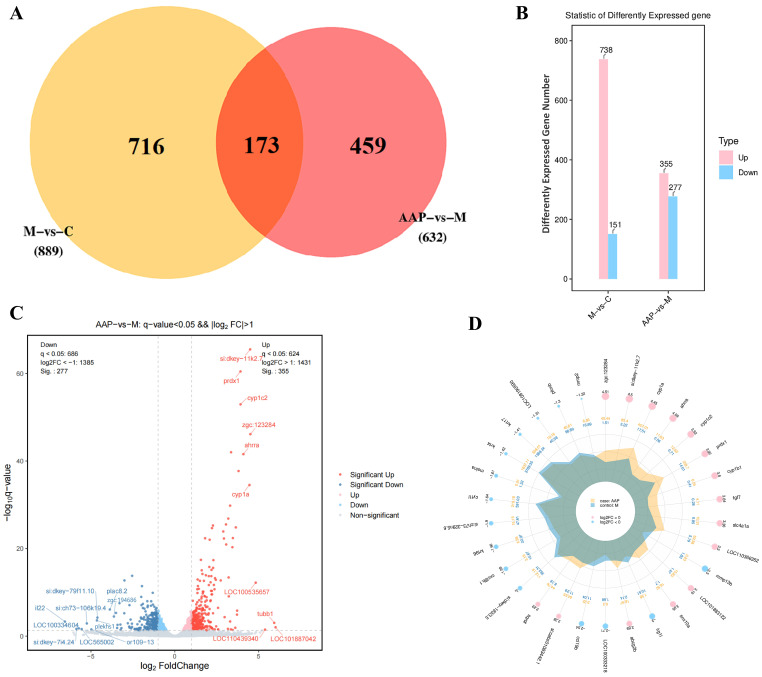
Effects of 2-AAP on genes in zebrafish. (**A**) Venn diagram. (**B**) Differential expression gene statistics. (**C**) Volcano plots of 2-AAP-vs.-M. (**D**) Differential gene radar map of 2-AAP-vs.-M. The first circle from outside to inside represents upregulated genes (red) and downregulated genes (blue), and the size of the circle varies according to the ∣Log2FC∣ value. The data in the second outer circle represent the average expression of the 2-AAP group. Genes that were upregulated in expression after drug administration are shown as yellow spikes in the graph.

A sequencing analysis was performed via high-throughput techniques to investigate differential gene expression at the transcriptome level following the administration of 2-AAP. A total of 889 genes were differentially expressed between the control and model groups, comprising 738 upregulated genes and 151 downregulated genes. Additionally, a total of 632 genes were differentially expressed between the model and 2-AAP groups, including 355 upregulated genes and 277 downregulated genes. A total of 173 differentially expressed genes were identified as common to the control, model, and 2-AAP groups (Figure 7A,B).

Ranked by ∣Log2FC∣, the top 10 most differentially downregulated genes were *plac8.2*, *zgc:194686*, *si:dkey-79f11.10*, *si:ch73-106k19.4*, *plekhs1*, *or109-13*, *LOC565002*, *LOC100334604*, *si:dkey-7i4.24*, and *il22*. The top 10 most differentially upregulated genes were *si:dkey-11k2.7*, *prdx1*, *cyp1c2*, *zgc:123284*, *ahrra*, *cyp1a*, *LOC100535657*, *tubb1*, *LOC110439340*, and *LOC101887042* (Figure 7C). Moreover, the top 30 genes with the smallest q values are displayed in the differential gene expression radar plot in the comparison between the model and 2-AAP groups (Figure 7D). The top 10 differentially expressed genes, as determined by the q value, were as follows: *zgc:123284*, *si:dkey-11k2.7*, *cyp1a*, *ahrra*, *cyp1c2*, *prdx1*, *cyp1b1*, *fgf7*, *slc4a1a*, and *LOC110366352*.

#### 2.7.2. Analysis of Gene Enrichment

**Figure 8:**
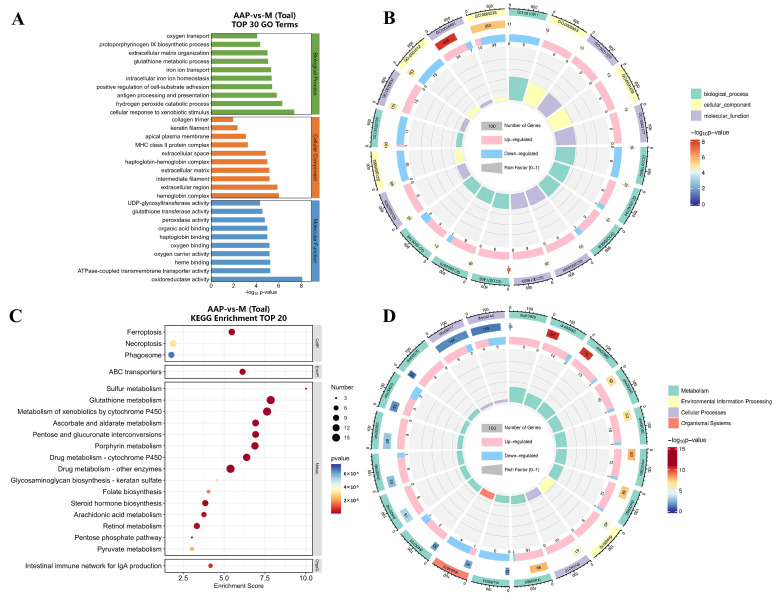
Enrichment analysis. (**A**) Top 30 GO terms in AAP-vs.-M. (**B**) GO analysis radar chart in AAP-vs.-M. The first circle represents the items of GO enrichment, and the outside of the circle represents a ruler with respect to gene number. Different colors represent different GO classifications. The second circle represents the number of genes enriched in the GO terms, and the color indicates the *p* value; the greater the number of genes, the longer the bar, and the smaller the *p* value, the redder the color. The third circle represents gene expression trends. (**C**) Top 20 pathways enriched in the AAP-vs.-M comparison. (**D**) KEGG analysis radar chart of the AAP-vs.-M comparison.

For the AAP-vs.-M comparison group, a total of 1047 Gene Ontology (GO) terms were obtained. The top 30 GO terms are presented in Figure 8A. The enriched biological process (BP) terms included the hydrogen peroxide catabolic process, intracellular iron ion homeostasis and iron ion transport, and the glutathione metabolic process. The enriched cellular component (CC) terms included the hemoglobin complex and haptoglobin–hemoglobin complex. The enriched cellular molecular function (MF) terms included glutathione transferase activity, peroxidase activity, and oxidoreductase activity. The results of the GO enrichment analysis are reflected in the radargrams (Figure 8B). As illustrated, the number of entries for BP was the highest, followed by MF and then CC. The largest number of genes were enriched in the extracellular region, with a total of 953 genes. The genes associated with oxidoreductase activity presented the smallest *p* values.

In the AAP-vs.-M comparison group, 113 KEGG pathways were obtained, and the top 20 KEGG pathways are presented in Figure 8C. 2-AAP was enriched in most metabolic pathways, including glutathione metabolism and the metabolism of xenobiotics by cytochrome P450. The second pathway was related to cellular processes, of which ferroptosis was a significant component. The results of the KEGG enrichment analysis are reflected in the radargram (Figure 8D), which demonstrates that the pathways enriched in metabolism were the most abundant, followed by those enriched in cellular processes. The largest number of genes, 194, was enriched in the cellular necroptosis pathway, whereas the glutathione metabolism pathway under metabolic classification had the smallest *p* value. These findings prompted us to investigate the potential roles of the ferroptosis and glutathione metabolic pathways in the alleviation of the atherosclerotic process by 2-AAP.

#### 2.7.3. GSEA

**Figure 9:**
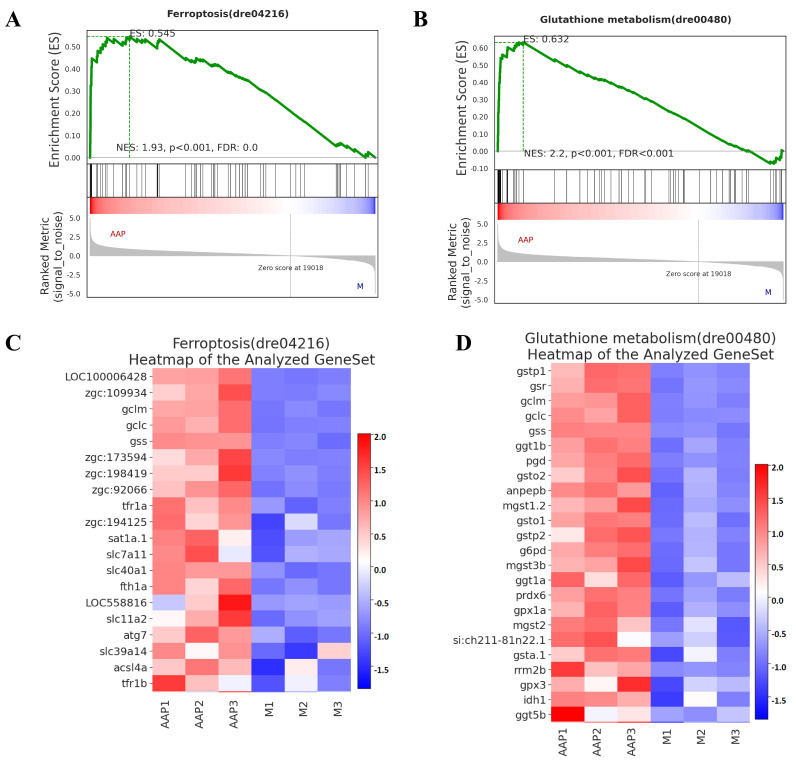
GSEA. (**A**) ES distribution map in ferroptosis. (**B**) ES distribution map of glutathione metabolism. (**C**) Differential gene-clustering heatmap for ferroptosis. (**D**) Differential gene-clustering heatmap of glutathione metabolism. The green line shows the distribution of ES for all genes. As depicted in the figure, when ES > 0, the gene on the left side of the dashed line is the core gene, which contributed significantly to the enriched pathways.

Ferroptosis can be attributed to the depletion of glutathione, a reduction in the activity of GPX4, and a decrease in the cellular antioxidant capacity. Differential genes that were enriched in the ferroptosis and glutathione metabolism pathways were analyzed separately via GSEA. As illustrated in the enrichment score distribution map (ES) (Figure 9A,B), NES > 1, *p* < 0.05, and FDR < 0.25 indicate that 2-AAP-interacting genes are significantly enriched in both pathways. In the ferroptosis and glutathione metabolism pathways, the core genes were significantly activated in the 2-AAP group. The core differentially expressed genes on the left of the peaks are displayed in the clustered heatmap (Figure 9C,D). Thus, 2-AAP may impact ferroptosis and glutathione metabolism by activating the expression of genes such as *gclm*, *gclc*, and *gss*.

#### 2.7.4. Effects of 2-AAP on Gene Expression

**Figure 10:**
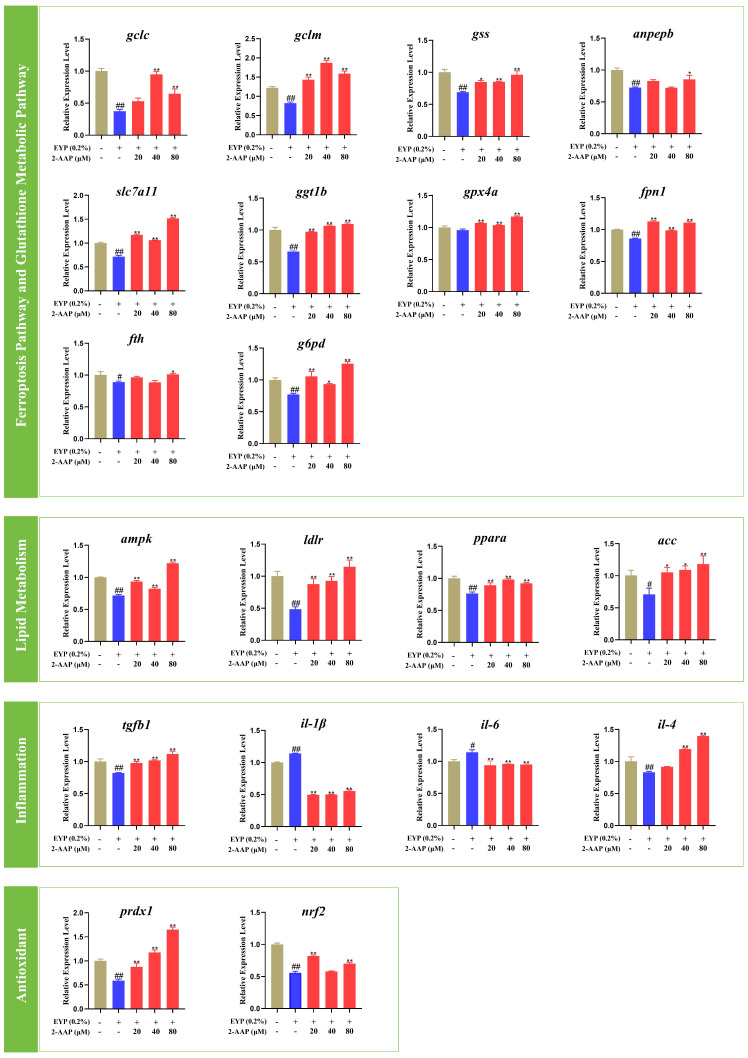
Expression of ferroptosis-, glutathione metabolism-, lipid metabolism-, inflammation-, and antioxidant-related genes following 2-AAP exposure. In the column chart, brown represents the blank group, blue represents the model group, and red represents the administration group. Compared with the control group, # *p* < 0.05, ## *p* < 0.01. Compared with the model group, * *p* < 0.05, ** *p* < 0.01.

Exposure to AAP resulted in notable alterations in the mRNA expression levels of genes associated with the ferroptosis and glutathione metabolism pathways. As depicted in Figure 10, the expression levels of *gclc*, *gclm*, *gss*, *anpepb*, *slc7a11*, *ggt1b*, and *gpx4*, which are related to GSH synthesis and transportation, were notably elevated. With respect to the genes involved in the storage and transportation of iron ions, the expression levels of *fpn1*, *fth*, and *g6pd* were increased in zebrafish treated with 2-AAP. Furthermore, our findings revealed that 2-AAP markedly influenced the mRNA expression levels of genes associated with lipid metabolism, inflammation, and oxidative stress. In zebrafish exposed to 2-AAP, the expression levels of pivotal genes involved in lipid metabolism, such as *ampk*, *ldlr*, *ppara*, and *acc*, were notably elevated. The mRNA levels of the proinflammatory genes *il-1β* and *il-6* substantially decreased. Conversely, the mRNA levels of the anti-inflammatory genes *tgfb1* and *il-4* markedly increased. Compared with those in the model group, the mRNA levels of the antioxidant-related genes *prdx1* and *nrf2* were notably elevated.

## 3. Discussion

When a large amount of fat is ingested, the amount of lipid metabolism in the body is lower than the amount ingested, and a large amount of lipids are not metabolized, resulting in lipid metabolism disorders, which are the basis of AS lesions [23]. Hyperlipidemia, inflammation, and oxidative stress contribute to the onset and progression of AS [24,25]. We simulated high-fat intake by feeding zebrafish egg yolk powder, and this experiment revealed that 2-AAP was able to improve lipid metabolism and alleviate inflammation and oxidative stress. Moreover, we found that 2-AAP was able to delay ox-LDL-induced macrophage differentiation into foam cells by reducing oxidized lipid uptake, relieving oxidative stress, and attenuating Fe^2+^ accumulation. Mechanistically, we found that 2-AAP exerts its anti-AS effects mainly by affecting the ferroptosis pathway and glutathione metabolism pathway. In addition, 2-AAP was also able to regulate key genes for lipid metabolism, inflammation, and oxidative stress, as shown by the PCR results, suggesting that the anti-AS activity of 2-AAP may be achieved through multiple pathways.

Zebrafish organs function similarly to those of mammals, with highly conserved gene expression and regulation, and could serve as a new model for studying human diseases [26]. At present, studies have shown that diet-induced hyperlipidemia in zebrafish shares pathophysiological pathways with those in mammals and is highly similar in terms of the molecular mechanisms (including lipid metabolism and lipoprotein oxidation) associated with atherosclerosis [27]. The small size and transparency of zebrafish allow experiments using zebrafish to combine lipid metabolism phenotyping with biochemical assays, through which the anti-atherosclerotic activity of drugs can be assessed. However, there were some limitations in our experiments, in the detection of lipid levels, due to the difficulty of blood sampling in zebrafish. In the experiment, we chose the whole homogenate of zebrafish rather than blood samples for the experiments. This choice limited the clinical relevance of the results but still reflected the lipid-lowering activity of 2-AAP to some extent.

In the present study, zebrafish were fed a high-fat diet to establish a lipid metabolism disorder model. As expected, the blood flow rate of zebrafish decreased dramatically, a large amount of lipids were deposited in the blood vessels, and, at the same time, the high-fat model of zebrafish presented inflammation and oxidative stress, which was highly consistent with the pathogenesis of early AS in mammals. In addition, the indicators of relevant AS were alleviated after 2-AAP administration, suggesting that the anti-AS effect of 2-AAP may be related to its lipid-lowering, anti-inflammatory, and antioxidant effects.

Some studies have shown that ox-LDL, a key lipoprotein in the pathogenesis of AS, can simulate the early formation of atherosclerotic plaques by inducing macrophages via ox-LDL [28]. Under normal conditions, LDL consists of triglycerides and cholesterol esters, with an outer layer encapsulated by phospholipids, free cholesterol, and apolipoprotein B (ApoB) [29]. Under pathological conditions, redox imbalance occurs, ApoB-containing lipoproteins are oxidized by ROS in the vascular intima, and ox-LDL is deposited in the subendothelial space of the vessel wall, which further contributes to endothelial rupture [30]. T cells, especially T-helper (Th1) cells, activate macrophages and exacerbate vascular injury by secreting cytokines, such as interferon gamma (IFN-γ) [31]. Massive numbers of macrophages accumulate in the intima and phagocytose ox-LDL to form foam cells while continuing to drive the inflammatory response and further accumulation of immune cells by releasing pro-inflammatory factors and reactive oxygen species [32]. Subsequently, smooth muscle cells shift from a contractile to a synthetic phenotype in response to inflammation or vascular endothelial injury, proliferate and migrate to the intima, and encapsulate foam cells and lipids. Additionally, early plaques eventually appear [31,33]. Macrophage phagocytosis of oxidized lipids and differentiation into foam cells are key steps in the complete process of AS plaque formation and fragmentation [34]. Therefore, we chose the model of the ox-LDL-induced differentiation of RAW264.7 cells into foam cells to investigate the activity of 2-AAP in slowing down plaque formation. After the phagocytosis of ox-LDL by RAW264.7 cells, many red particles were observed in the cells, and the intracellular lipid content was increased. Treatment with 2-AAP alleviated RAW264.7 cell phagocytosis of ox-LDL; reduced the intracellular TC, TG, FC, and CE contents; and decreased the CE/TC ratio, demonstrating that 2-AAP inhibited foam cell formation and slowed the process of atherosclerotic plaque formation. In addition to macrophages, some other cell types, such as smooth muscle cells and endothelial cells, also play important roles in atherosclerotic plaque formation. Whether 2-AAP can alleviate the lesion of these cells requires more study in the future.

The analysis of the mechanism of 2-AAP anti-AS at the gene level through the transcriptome revealed that 2-AAP was able to significantly affect the expression of the antioxidant gene prdx1, indicating that prdx1 may be an important target of 2-AAP anti-AS. The top 30 GO terms enriched for AS-related genes were related mainly to iron homeostasis and redox, indicating that the main direction of action of 2-AAP is to maintain iron homeostasis and redox homeostasis. Among the top 20 pathways analyzed by KEGG, ferroptosis and glutathione metabolism are associated with redox reactions. Arachidonic acid metabolism is closely associated with inflammation. Differential gene expression in the ferroptosis and glutathione metabolism pathways was particularly significant, indicating that 2-AAP may exert its antioxidative stress effects through these two pathways.

Ferroptosis promotes the progression of atherosclerosis [35]. An imbalance in intracellular iron homeostasis leads to excessive levels of ferrous ions, and excess Fe^2+^ induces cellular oxidative stress via the Fenton reaction, resulting in elevated intracellular ROS levels [36]. Research has shown that GPX4 plays an important role in the pathophysiological processes of AS by inhibiting ferroptosis [14]. GPX4, a strong reductase in cells, reduces oxidative damage in endothelial cells and is an important predictor of AS [37]. Ox-LDL treatment resulted in elevated levels of intracellular ferrous ions, accompanied by elevated levels of MDA and ROS and decreased GPX4 viability. What is more, we found that 2-AAP regulated ferroptosis and glutathione metabolic pathways, reduced the content of Fe^2+^, MDA, and ROS in cells, and increased GPX4 viability. 2-AAP also increased the expression of genes related to GSH synthesis and ferrous ion transport. These results indicated that 2-AAP might exert anti-AS activity by targeting ferroptosis pathway. Therefore, we hypothesized that 2-AAP had a potential effect to regulate ferroptosis in anti-atherosclerosis. Medications commonly used in the treatment of atherosclerosis typically include lipid-lowering agents, antiplatelet agents, and antihypertensives, which primarily target symptoms and risk factors [14]. Ferroptosis accelerates endothelial dysfunction and lipid peroxidation by affecting the state of endothelial cells, macrophages, and smooth muscle cells, providing a new target for AS [13]. Additionally, 2-AAP, which affects ferroptosis, offers a potential treatment for the disease.

Concurrently, the expression levels of other AS-related genes were also investigated in this study, and 2-AAP was found to increase the expression of lipid metabolism-, anti-inflammatory-, and redox-related genes, suggesting that the anti-AS effect of 2-AAP may involve multiple pathways.

## 4. Materials and Methods

### 4.1. Chemicals and Reagents

2-acetamidophenol was purchased from Macklin Co., Ltd. (Shanghai, China). Egg yolk powder was purchased from Yuanye Biotech Co., Ltd. (Shanghai, China). 2-propanol, 1,2-propanediol, and Tween 20 were purchased from Sinopharm Chemical Reagent Co., Ltd. (Shanghai, China). Oxidized human LDL (ox-LDL) was purchased from Yiyuan Biotech Co., Ltd. (Guangzhou, China). TCH/T-CHO, TG, LDL-C, HDL-C, MDA, T-SOD, CAT, and T-AOC assay kits were purchased from Nanjing Jiancheng Bioengineering Institute Co., Ltd. (Nanjing, China). The SPARKeasy Improved Tissue/Cell RNA Kit was purchased from SparkJade Biotech Co., Ltd. (Jinan, China). HiScript^®^ II Q RT SuperMix for qPCR (+gDNA wiper) and ChamQ Universal SYBR qPCR Master Mix were purchased from Vazyme Biotech Co., Ltd. (Nanjing, China).

### 4.2. Zebrafish Maintenance

The zebrafish used for the experiments were provided by the Engineering Research Center of Zebrafish Models for Human Diseases and Drug Screening in Shandong Province (Jinan, China). All adult zebrafish were maintained under a constant photoperiod cycle of 14 h light/10 h dark at 28 ± 0.5 °C in an automated zebrafish rearing system (ESEN, Beijing, China). The zebrafish were fed live brine shrimp twice daily.

On the day before mating, healthy and sexually mature zebrafish were placed in a spawning tank with a partition in the middle. At the beginning of the next light cycle, the partition was removed, and the embryos were collected 2 h later. Zebrafish embryos were disinfected and washed before being transferred to zebrafish culture water (0.4 mM CaCl_2_, 5 mM NaCl, 0.16 mM MgSO_4_, and 0.17 mM KCl) containing 0.1% methylene blue. All experiments were carried out in compliance with standard ethical guidelines and under the control of the Biology Institute, Qilu University of Technology Animal Ethics Committee (SWS20220929).

### 4.3. Zebrafish Grouping and Drug Treatment

Five days post fertilization (dpf), wild AB zebrafish were selected for randomization into control, model, positive, and 2-AAP-treated groups, with 3 parallel wells of 20 fish per group. Zebrafish in the control group were cultured in zebrafish culture water, and zebrafish in the model, positive, and 2-AAP-treated groups were fed 0.2% egg yolk powder to establish a zebrafish hyperlipidemia model. After being fed egg yolk powder for 48 h, the control and model groups were cultured in zebrafish culture water. The positive control group was treated with AC (Aladdin Biotech Co., Ltd., Shanghai, China) at a final concentration of 0.4 μM, whereas the 2-AAP-treated group was treated with 2-AAP at final concentrations of 20, 40, and 80 μM. The culture medium of each group of zebrafish was changed every 24 h, and the zebrafish were cultured in a constant-temperature incubator at 28 °C for a total of 48 h.

### 4.4. Oil Red O Staining

Oil Red O staining was used to analyze lipid deposition in zebrafish blood vessels. The Oil Red O (Sigma-Aldrich Co., Ltd., Shanghai, China) staining method was as follows. The treatment solution for the zebrafish was removed, PFA was added, and the mixture was fixed for 4 h at room temperature or overnight at 4 °C. Sequentially, 25%, 50%, 75%, and 100% of 1,2-propanediol/PBS were applied for 10 min for dehydration. Oil Red O in 0.6% staining solution was used for staining at 28 °C for 4 h. Sequentially, 100%, 75%, 50%, and 25% 1,2-propanediol/PBS were used for decolorization for 10 min. A PBS solution containing 0.1% Tween 20 was used to elute for 15 min × 4 times. Zebrafish were fixed with 4.2% methylcellulose and photographed from the side view under a Zeiss fluorescence microscope (Carl Zeiss AG, Oberkochen, Germany) to observe the lipid staining of the blood vessels in the tails of the zebrafish. Lipid lowering rate (%) = (IOD of the model group-IOD of the 2-AAP-treated group)/IOD of the model group × 100%.

### 4.5. Biochemical Analysis

After each group of zebrafish was incubated in a constant-temperature incubator at 28 °C for 48 h, the treatment solution of each group was removed, and the zebrafish in each group were washed three times with PBS. Each zebrafish was transferred to a 1.5 mL centrifuge tube, any liquid remaining in the tube was removed, the mixture was accurately weighed, and PBS was added at a ratio of weight (g):volume (mL) = 1:9. The tissue homogenate was prepared by grinding at 10,000–15,000 revolutions per minute (rpm) via a tissue cell crusher and was subsequently centrifuged at 3000 rpm for 15 min at 4 °C, after which the supernatant was analyzed immediately. Protein concentrations and T-CHO, TG, LDL-C, and HDL-C levels were determined according to the kit instructions.

### 4.6. Mean Blood Flow Velocity Measurement

Zebrafish were fixed with 4.2% methylcellulose, and videos were taken at the side view angle for 15 s. The average blood flow velocity was detected using zebrblood^TM^ v 1.3.2(ProBeCare Co., Ltd, Wuhan, China), and the average blood flow velocity of the zebrafish tail blood vessels was calculated.

### 4.7. Macrophage Aggregation Assay

The positive control drug selected was indomethacin (IMC), which is recognized to have good anti-inflammatory activity. Macrophage fluorescent *Tg(lyz:EGFP)* zebrafish were selected for the macrophage aggregation experiments described in Section 4.3. *Tg(lyz:EGFP)* zebrafish were fixed with 4.2% methylcellulose and photographed at the side view angle, and the macrophage accumulation status of the zebrafish tail was observed under an Olympus fluorescence microscope (Olympus Corporation, Tokyo, Japan).

To visualize macrophage aggregation in blood vessels, green vascular fluorescent *Tg(fli1:EGFP)* zebrafish males were crossed with macrophage red fluorescent *Tg(lyz:DsRed2)* females, and, after zebrafish embryos were obtained, experiments were carried out as described in Section 4.3.

### 4.8. Antioxidant Capacity Test

After each group of zebrafish was incubated in a constant-temperature incubator at 28 °C for 48 h, the treatment mixture was removed from each group. DCFH-DA (a probe for the detection of reactive oxygen species) at a final concentration of 10 μM was added to each group and incubated for 30 min at 28 °C in the dark. The zebrafish were subsequently washed three times with PBS. Each group of zebrafish was photographed at the side view angle under a Zeiss fluorescence microscope.

The T-SOD activity, CAT activity, T-AOC, and MDA content were detected using a reagent kit, and the sample preparation was the same as that described in Section 4.5.

### 4.9. Cell Culture

Mouse macrophage RAW264.7 cells were purchased from Pricella Life Technology Co., Ltd. (Wuhan, China). RAW264.7 cells were cultured in DMEM with 10% fetal bovine serum (AusGeneX, Brisbane, Australia) and 1% penicillin-streptomycin solution (Pricella Life Technology Co., Ltd., Wuhan, China). The number of cell generations used in the experiment was within 10 generations. The cells were placed in a 37 °C constant-temperature incubator containing 5% CO_2_ for cultivation.

### 4.10. Cell Grouping and Drug Treatment

When the density of RAW264.7 cells reached 70–80%, RAW264.7 cells with better growth status were inoculated into 96-well plates at a density of 1 × 10^5^ cells/mL in 100 μL per well. RAW264.7 cells were divided into control, model, and 10, 20, and 40 μM 2-AAP groups, with 3 replicates in each group. After the cells adhered to the wall, they were washed twice with PBS, and the cells were starved with serum-free DMEM for 12 h and then exposed to serum-containing culture medium for 3 h. Cells pretreated in this way were synchronized with respect to the cell cycle. The control cells were cultured in serum-containing culture medium, ox-LDL was added to the model cells at a final concentration of 75 μg/mL, and 75 μg/mL ox-LDL and 10, 20, and 40 μM 2-AAP were added to the 2-AAP-treated cells, which were cultured for 24 h.

### 4.11. Cell Viability Assay

After RAW264.7 cells were subjected to cycle synchronization, they were exposed to different concentrations of 2-AAP (10, 20, 40, 80, 160, 320, or 640 μM) for 24 h. CCK-8 solution (100 μL of DMEM containing 10 μL of CCK-8) was added (100 μL per well) and incubated for 30 min. According to the CCK-8 kit instructions, the OD value of each well was measured at 450 nm with a SPECTROstar Nano (BMG Labtech, Offenburg, Germany) to calculate the cell viability and assess drug safety.

### 4.12. Cell Oil Red O Staining

Each group of RAW264.7 cells was cultured under suitable conditions for 24 h and then stained with Oil Red O. The cells were washed twice with PBS and then treated with PFA for 20–30 min at room temperature, followed by 60% isopropanol for 20–30 s. The 0.6% Oil Red O staining solution was prepared with isopropanol for 10–20 min at room temperature, and then 60% propylene glycol was added for 20–30 s. After washing with distilled water 2–5 times, each well was covered with 100 μL of distilled water and photographed under white light with an Olympus inverted microscope.

### 4.13. Intracellular Lipid Assay

Drug treatment of RAW264.7 cells was carried out in T25 cell culture flasks, and, after 24 h of drug administration, the groups of cells were collected in 1.5 mL centrifuge tubes using a spatula. The cells were lysed according to the method recommended in the kit instructions, and the supernatant was collected for testing. The TC, FC, CE, and CE/TC ratios were determined using the Amplex Red Cholesterol and Cholesteryl Ester Assay Kit (Beyotime Biotechnology Co., Ltd., Shanghai, China). A TG assay kit was used to determine the TG content. The protein concentrations were determined using BCA kits, and TC, TG, and CE contents were measured per milligram of protein in each group.

### 4.14. Detection of Ferrous Iron

After 24 h of treatment in each group, serum-free DMEM containing 1 μmol/L FerroOrange (Dojindo, Kumamoto, Japan) was added to the wells, and the samples were incubated at 37 °C in 5% CO_2_ for 30 min and finally photographed with an ImageXpress Micro Confocal system (Molecular Devices Co., Ltd, Shanghai China).

### 4.15. Detection of ROS

The intracellular ROS content was detected via a DCFH-DA Reactive Oxygen ROS Fluorescent Probe (Solarbio Biotech Co., Ltd., Beijing, China). After 24 h, serum-free DMEM containing 10 μM DCFH2-DA was added to the wells, which were incubated at 37 °C in 5% CO_2_ for 30 min and photographed via high-content screening.

### 4.16. Detection of GPX4 and MDA

After 24 h of treatment with 2-AAP, the RAW264.7 cells were collected and sonicated in an ice-water bath. The supernatant was collected after centrifugation for testing. The cells in each group were collected and assayed according to the MDA kit method, and GPX4 activity was detected using the ELISA kit double-antibody sandwich method (Meimian Industrial Co., Ltd.,Yancheng, China).

### 4.17. RNA Sequencing

The treatment solution of each group was removed, and the zebrafish in each group were washed three times with RNase-free water and transferred to 1.5 mL centrifuge tubes. The entire RNA-Seq sequencing and analysis process was performed by Oebiotech Co., Ltd. (Shanghai, China).

### 4.18. RT-qPCR

The total RNA of the zebrafish samples from each group was extracted according to the instructions of the SPARKeasy Improved Tissue/Cell RNA Kit, and the RNA was reverse transcribed into cDNA using HiScript^®^ II Q RT SuperMix for qPCR (+gDNA wiper). The quantitative polymerase chain reaction (qPCR) system was prepared according to the recommended ChamQ Universal SYBR qPCR Master Mix system. The thermal cycling program on the LightCycler^®^96 instrument was 95 °C for 1 min, followed by denaturation at 95 °C for 20 s per cycle, annealing at 55 °C for 20 s, and extension at 72 °C for 30 s for a total of 45 amplification cycles. Rpl13a was used as an internal reference gene. The differences in gene expression were determined via the 2^−ΔΔCt^ method. The sequences of primers used in the study are shown in Table 1.

### 4.19. Statistical Analysis

The results for each group are presented as the mean ± SEM. Differences between multiple groups were tested via one-way ANOVA, and *p* < 0.05 was considered to indicate a statistically significant difference.

## Figures and Tables

**Table 1:** Primer sequences.

Genes	Forward	Reverse
*rpl13a*	5′-CGGTCGTCTTTCCGCTATTG-3′	5′-CTTGCGGAGGAAAGCCAAAT-3′
*gclc*	5′-CTCCTCACAGTCACGGCATT-3′	5′-TCTTGGGTGTCGGTTGATGG-3′
*gclm*	5′-TTTCAGYCATGCGACAACGC-3′	5′-TTTCAGTCATGCGACAACGC-3′
*gss*	5′-ACATGGAGTGTTGATGCGGA-3′	5′-TGCCAGTGCCTGATGAAAGA-3′
*anpepb*	5′-GCCACAATCATCGCCCTTTC-3′	5′-GTTGGGATGTTTGTCGTCGC-3′
*slc7a11*	5′-GGACGATCATTGGAGCAGGA-3′	5′-ACACCACCAGTGACATTCCC-3′
*ggt1b*	5′-ATCACCTCCAAAGGCTACGC-3′	5′-GGGAAGGTAAAACTCGGGCT-3′
*gpx4a*	5′-CATCCTGGCTTTCCCTTCCA-3′	5′-CCCGTCGCCATTCACATCAA-3′
*fpn1*	5′-CTTGACCGTGACCCAACTGA-3′	5′-GCCACGAAGGAAACGGAGAT-3′
*fth*	5′-GCTTTCTACTTTGACCGGGAC-3′	5′-AATGCGTCCACTCTCTTGT-3′
*g6pd*	5′-CACACCTACTGTCTCTGCCTG-3′	5′-CATCATGGTAGACGCTGGGT-3′
*ampk*	5′-CAGGAACCGCTACACCTCAC-3′	5′-GAGCCTTCCGCCACTTTACT-3′
*ldlr*	5′-ATATCACAACGGACGAG-3′	5′-ACAAGTATTTCAGCCAC-3′
*pparα*	5′-GTTCGTCAGGGGAATGGAGG-3′	5′-CGGACTGGTTCTCGGTCATC-3′
*acc*	5′-GGGCAATCATCCGTCACTCA-3′	5′-TCCTCAATCTTTGAAGGGTCCA-3′
*tgfb1*	5′-GTCCGAGATGAAGCGCAGTA-3′	5′-TGGAGACAAAGCGAGTTCCC-3′
*il-1β*	5′-CATTTGCAGGCCGTCACA-3′	5′-GGACATGCTGAAGCGCACTT-3′
*il-6*	5′-TCAGCACTCCTCTCCTCAAA-3′	5′-ATCCATCTCTCCGTCTCTCAC-3′
*il-4*	5′-GCATATACCGGGACTGGAAACTG-3′	5′-CACATGTTCTTATGTCCTTTGCGCC-3′
*prdx1*	5′-TGGTGCTTCTGTCGATTCCC-3′	5′-CTGCCACCAGAGGGACATTC-3′
*nrf2*	5′-TCCAAACACCAGCTCAACGA-3′	5′-CGGACTTTGTTCTTGCCTCG-3′

## Data Availability

The data presented in the current study are available on request from the corresponding author.
